# No advantage for remembering horizontal over vertical spatial locations learned from a single viewpoint

**DOI:** 10.3758/s13421-017-0753-9

**Published:** 2017-09-05

**Authors:** Thomas Hinterecker, Caroline Leroy, Mintao Zhao, Martin V. Butz, Heinrich H. Bülthoff, Tobias Meilinger

**Affiliations:** 1 Max-Planck-Institute for Biological Cybernetics, Spemannstr. 38, D-72076 Tübingen, Germany; 2 Eberhard-Karls University, Tübingen, Germany

**Keywords:** Spatial memory, Horizontal, Vertical, Orientation dependency

## Abstract

Previous behavioral and neurophysiological research has shown better memory for horizontal than for vertical locations. In these studies, participants navigated toward these locations. In the present study we investigated whether the orientation of the spatial plane per se was responsible for this difference. We thus had participants learn locations visually from a single perspective and retrieve them from multiple viewpoints. In three experiments, participants studied colored tags on a horizontally or vertically oriented board within a virtual room and recalled these locations with *different layout orientations* (Exp. 1) or from *different room-based perspectives* (Exps. 2 and 3). All experiments revealed evidence for equal recall performance in horizontal and vertical memory. In addition, the patterns for recall from different test orientations were rather similar. Consequently, our results suggest that memory is qualitatively similar for both vertical and horizontal two-dimensional locations, given that these locations are learned from a single viewpoint. Thus, prior differences in spatial memory may have originated from the structure of the space or the fact that participants navigated through it. Additionally, the strong performance advantages for perspective shifts (Exps. 2 and 3) relative to layout rotations (Exp. 1) suggest that configurational judgments are not only based on memory of the relations between target objects, but also encompass the relations between target objects and the surrounding room—for example, in the form of a memorized view.

**Electronic supplementary material:**

The online version of this article (10.3758/s13421-017-0753-9) contains supplementary material, which is available to authorized users.

Knowing where things are in space is a fundamental cognitive ability. For instance in everyday life we are often required to recall the locations of objects on a table or within a shelf. Unsurprisingly a fair amount of research has already dealt with determining the characteristics of such spatial long-term memory (see McNamara, Sluzenski, & Rump, 2008 and Waller & Greenauer, 2013 for reviews). Much of this research focused on locations distributed in the horizontal plane and neglected comparisons with the vertical plane. When researchers have conducted such comparisons the results showed an advantage in memory for horizontal as compared to vertical space (see Jeffery, Jovalekic, Verriotis, & Hayman, 2013b for a review). However such a horizontal advantage in spatial memory was found only when spatial memory was acquired through navigation. In the present study we investigated whether the horizontal advantage in spatial memory is bound to a space that was acquired through navigation, or whether it is a general property of spatial memory and can therefore be observed in spaces that were not learned through navigation but instead were perceived from a single perspective.

The horizontal advantage in spatial memory was advocated by Jeffery et al.’s (2013b) bicoded-map hypothesis. According to this hypothesis, surface-travelling mammals possess a spatio-cognitive system that is tuned to two-dimensional surface-bound navigation and treats three-dimensional space asymmetrically. The term “bicoded” refers to the asymmetrical encoding of three-dimensional space (i.e., space is not represented uniformly along horizontal and vertical planes). This hypothesis is based on empirical findings concerning neural mechanisms involved for representing navigable spaces (i.e., space that a navigator can move through). Representations of such navigable spaces are assumed to rely on grid (Hafting, Fyhn, Molden, Moser, & Moser, 2005) and place cells (O’Keefe & Dostrovsky, 1971) located in entorhinal and hippocampal cortical structures, respectively. It was shown with rats that these types of cells work asymmetrically across the spatial planes, with a more fine-grained resolution for horizontal than vertical space (Hayman, Verriotis, Jovalekic, Fenton, & Jeffery, 2011).

Although evidence supporting the horizontal advantage in spatial memory comes primarily from experiments with nonhuman animals (see also Jeffery, Wilson, Casali, & Hayman, 2015), human navigation behavior exhibits similar horizontal advantage in spatial memory. For instance, people seem to encode or recall horizontal locations in a building more accurately than vertical ones (Luo, Luo, Wickens, & Chen, 2010; Montello & Pick, 1993; Tlauka, Wilson, Adams, Souter, & Young, 2007; Wilson, Foreman, Stanton, & Duffy, 2004). Furthermore, people who are experienced with a particular building seem to organize their memory of this building along the horizontal dimension (i.e., floors) rather than along the vertical dimension or along no specific dimension at all (Hölscher, Meilinger, Vrachliotis, Brösamle, & Knauff, 2006). Humans also show a more accurate memory of a novel building when they took a floor-by-floor strategy than when learning it along vertical columns (Thibault, Pasqualotto, Vidal, Droulez, & Berthoz, 2013). Recently, a study added to these findings by showing that participants memorized a building by floors regardless of the learning strategy (Dolle, Droulez, Bennequin, Berthoz, & Thibault, 2015). In addition, human memory seem to be exhibit greater vertical than horizontal distortions regarding the size of familiar buildings (Brandt et al., 2015).

Although the aforementioned studies suggest that human memory of vertical and horizontal locations are qualitatively different (hereafter termed as *horizontal superiority hypothesis*), they cannot differentiate what may underlie such memory difference. Specifically, since spatial memory was acquired via actual navigation/exploration in these studies, it remains unclear whether the observed horizontal superiority in spatial memory is caused (1) by different organization of spatial memory of vertical and horizontal planes in general or (2) by different ways of navigating through the environment. To differentiate between these possibilities, we asked our participants to learn vertical or horizontal spatial relations from a single perspective rather than via navigation. If spatial memory varies as a function of spatial plane orientation generally, we should observe the horizontal superiority in overall performance. Alternatively, if navigational experience is crucial for observing this superiority, spatial memory acquired from a single perspective should not vary between horizontal and vertical planes.

Previous studies on spatial memory for object locations and spatial relations that are perceivable from a single vantage point were often implemented as perspective change paradigms. That is, participants learned a layout of spatial locations from one viewpoint and then recognized the layout or recalled the spatial relations from different perspectives. Although previous studies have shown that memory recall varies with perspective change around the horizontal plane, whether perspective change would have the same influence on spatial memory of vertical locations and with vertical viewpoint shifts remains unknown.

Spatial memory of horizontal locations is typically orientation-dependent. That is, retrieving location information is easier from the viewpoint(s) taken during the time of learning (often referred to as 0° viewpoint orientation), but slower and more prone to errors for novel perspectives, which differ in their orientation in relation to the experienced view(s) (e.g., by ± 135°) (e.g., Christou & Bülthoff, 1999; Diwadkar & McNamara, 1997; Shelton & McNamara, 1997). Importantly, numerous studies showed that performance for some nonexperienced viewpoints can be better than for others (e.g., Kelly & McNamara, 2008; Meilinger & Bülthoff, 2013; Shelton & McNamara, 2001). This phenomenon usually occurs in experimental setups in which the to-be-studied object location layout possesses salient intrinsic axes resulting from orthogonality and/or symmetry of the layout (e.g., when it comprises of rows and columns). Under such circumstances, recall performance is not simply decreasing with increasing orientation discrepancy of a novel perspective with respect to the experienced one, but depends on whether or not the orientation of the taken perspective is parallel with an intrinsic axis of the layout (e.g., Mou & McNamara, 2002). For instance, memory recall from novel perspectives that are contra-aligned (shifted by 180°) or orthogonally aligned (shifted by ± 90°) with respect to the encoded view can be better than recall from nonexperienced oblique perspectives (e.g., shifted by ± 45° or ± 135°) and can even approach the performance for the encoded view (e.g., Kelly & McNamara, 2008).

The better performance for contra-aligned perspectives observed in horizontal spatial memory might not apply to spatial memory of vertically distributed locations. More specifically, we propose a *horizontal contra-alignment advantage hypothesis*. Whereas the contra-aligned perspective is privileged in the horizontal plane—meaning that recall performance from this perspective is better than recall from other novel perspectives—no such contra-aligned advantage might occur in the vertical plane. This hypothesis is motivated by the fact that humans scarcely see a spatial scene from a full-body upside down perspective due to the force of gravity and struggle with imagining such viewpoints, as informal reports suggest (Waller, 2014). The advantage for contra-aligned horizontal perspectives might be because humans have much more experience with such perspectives, as they can walk around in the horizontal plane. In addition, humans often mentally take contra-aligned perspectives in the horizontal, but not in the vertical plane. For instance, humans often take the spatial perspective of another person in interpersonal interactions (Cavallo, Ansuini, Capozzi, Tversky, & Becchio, 2017; Mainwaring, Tversky, Ohgishi, & Schiano, 2003; Schober, 1993; Tversky, Lee, & Mainwaring, 1999), which most likely take place in the horizontal plane and often with persons facing them (contra-aligned to themselves). Conceivably, such a greater extent of experience in spatial perspective taking might improve the efficiency in transforming an encoded perspective to infer the spatial relations from nonexperienced contra-aligned perspectives during retrieval. If so, a lack of perspective taking experience in the vertical plane might cause the same physical transformation process to work less efficiently than in the horizontal plane.

Consequently, the contra-aligned perspective is privileged in the horizontal plane, meaning that the recall performance at horizontally distributed object locations for the contra-aligned orientation should be better than that at other novel orientations. In contrast, when recalling object locations distributed along the vertical plane, we expect that performance for the contra-aligned orientation will not be different from that for other novel orientations. Additionally, when comparing both spatial planes, we expect to observe better performance for the contra-aligned orientation in the horizontal plane. We expected no such advantage for other novel test orientations.

To test our predictions, we used a perspective change paradigm in which visual information was present not only during learning, but also during retrieval of the spatial relations (similar to Meilinger & Bülthoff, 2013). This required participants to recognize their current perspective and then recall a certain spatial relation. In addition, we implemented a paradigm in which participants had to recall the spatial relations not from varying perspectives, but with changing layout orientations (i.e., the layout was rotated). Conceivably, the aforementioned differences in memory recall might also apply for such an experimental setup, since participants are still required to recognize the layout orientation before recalling a certain spatial relation. As a result, in the case of the contra-aligned layout orientation, the spatial information should be used more efficiently in the horizontal plane.

In sum, we tested whether there is a general advantage in spatial memory of horizontal relative to vertical locations (i.e., the horizontal superiority hypothesis) when these locations were not learned by navigation but perceived from a single perspective. Furthermore, we examined whether perspective taking affects the recall of spatial memory of vertical and horizontal planes differently (i.e., the horizontal contra-aligned advantage hypothesis). We tested these hypotheses with three experiments. In Experiment 1 we manipulated retrieval by rotating the spatial layout (i.e., layout change), whereas in Experiments 2 and 3 we manipulated retrieval by rotating the whole environment (i.e., viewpoint change).

## Experiment 1

In this experiment we tested the horizontal superiority and horizontal contra-aligned advantage hypotheses, targeting recall level and patterns, respectively. Participants were required to recall learned spatial relations for different test orientations due to layout rotations.

### Method

#### Participants

Twenty-two naïve participants (11 women, 11 men), ages 20 to 54 years (*M* = 27.18, *SD* = 7.63), were recruited from a participant database in exchange for monetary compensation. All participants gave written informed consent before the experiment.

#### Materials

**Fig. 1:**
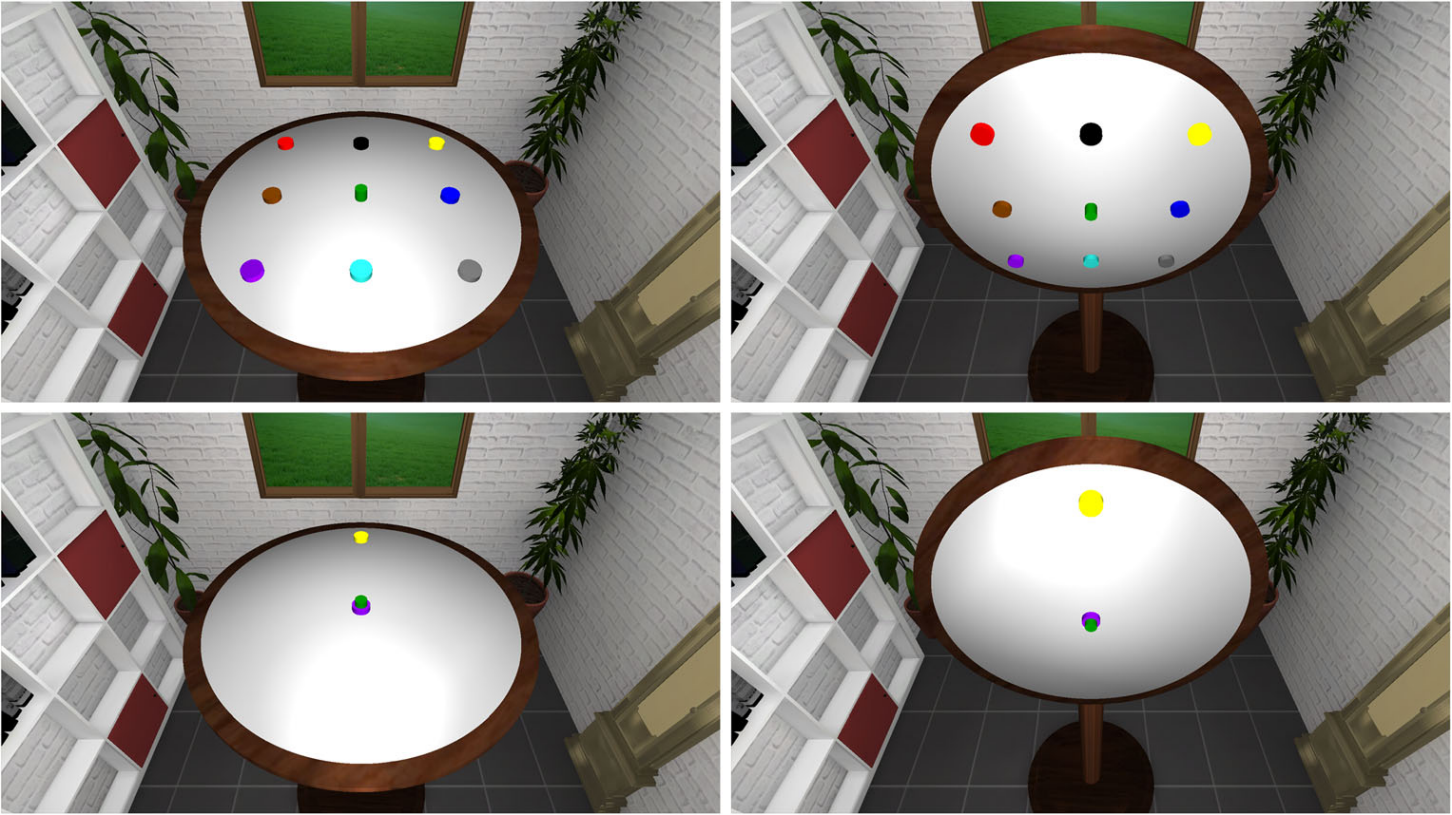
The virtual setup used in Experiment 1 for horizontal (left side) and vertical (right side) learning and testing. Participants learned a 3 × 3 object layout presented to them within a virtual room (top row). In a test trial, they always saw the center object, a second, reference object always located behind/above, and a target object, which they were asked to move to its correct location (bottom row). Participants were tested with different board orientations while remaining at the same position in the room. The present example corresponds to a board orientation of 45°. The correct target location is at the bottom. In Experiments 2 and 3, the whole room was rotated, and therefore the participants’ viewpoint in the room varied

The experiment comprised learning and testing phases for horizontal and vertical locations, respectively. During these phases, participants saw a cubical room (2.2 × 2.2 × 2.2 m) within a virtual reality (VR) display. This room was furnished with a shelf, a grandfather clock, a window, some plants, and a door (Fig. 1). This rendered all walls, and therefore the orientation of or position within the room, easily distinguishable for participants. We positioned a circular board, 75 cm in diameter and attached to the ground by a table leg, at the center point of this room either horizontally or vertically. Participants watched the room from a position 55 cm in front of and 55 cm above the center point of the board, corresponding to an eye height of 1.65 m. We chose this viewpoint to ensure that looking at the center of the table would yield the same average visual distortion with both horizontal and vertical board orientations.

During the learning phases (Fig. 1, top row), nine differently colored objects were attached to the board. These objects were arranged in a 3 × 3 grid, with a distance of 19.57 cm between the neighboring objects in each row and column. Eight of these objects were of the same cylindrical shape (4-cm diameter, 2-cm height). The object in the center, however, was smaller in diameter (2.7 cm) and higher (3 cm) than the other eight.

For testing (Fig. 1, bottom row), the board orientation varied by 0°, ± 45°, ± 90°, ± 135°, or 180° relative to the experienced board orientation, whereas participants remained at the same position in the room as during learning. Participants saw two reference objects, one of which was always the center object and the other of which was the object located above/behind the center object. This second reference object varied across board orientations but was always the same for a particular board orientation. Half a second later, the target stimulus appeared in the center of the board, as well. Since the shape of the center reference object differed from the shape of the other eight stimuli, both the target and the center reference object were visible, even though both occupied the center position of the board. Each of the eight board orientations was used once with each of the seven possible target objects. This resulted in 56 experimental test trials per learning condition. The test trials were presented in a random order for each participant. Participants operated the joystick of a gamepad (Logitech Rumble Gamepad F510) with their left thumb to move the target object to its correct location within the layout. They indicated the correct positioning with a buttonpress with their right thumb. After they had indicated the position the screen turned black, and the next trial started after 2 s.

We recorded the latency, defined as the time between the onset of the target stimulus and the buttonpress. In addition, we recorded the absolute angular error, defined as the difference between the correct direction of the target object from the center object and the direction that the target was placed at. We also analyzed absolute distance error, which yielded results almost identical to those for angular error.

The virtual experiment was created using Unity 3D (licensed version 4, Unity Technologies, San Francisco) and was presented using an Occulus Rift (Developmental Kit 1, Oculus VR, LCC) head-mounted display (HMD). The HMD has a resolution of 1,280 × 800 pixels for each eye, with 100% overlap of the eye images and an interpupillary distance set to 6.4 cm. Participants sat on a chair throughout the experiment. They were allowed to look around the room (i.e., also to their back), but they were asked to stay seated.

#### Design

The experiment consisted of a 2 × 8 × 2 mixed-factorial design. The first within-subjects factor was Horizontal or Vertical Plane (Fig. 1). The second within-subject factor was the Test Orientation of the Board, comprising eight different orientations (from 0° to 180° in ± 45° steps). For a better presentation of the different orientations concerning the horizontal contra-aligned advantage hypothesis, we combined the orientations between the orientation experienced during learning (0°) and the contra-aligned orientation (180°) and called these the *in-between orientations*. We ran the analyses with the combined orientations, but have also included the plots with all individual test orientations as Supplementary figures. The final between-subjects factor was the Sequence Order of the Plane Orientations During Learning (horizontal or vertical plane first). We counterbalanced the orders across participants.

#### Procedure

After oral and written instructions about the procedure of the study, we obtained written consent from the participants. Following this the HMD was adjusted individually, and participants could familiarize themselves with the VR. In each learning condition (horizontal or vertical plane), participants learned the color arrangement of the nine stimuli on the board for at least 3 min. We assigned the colors randomly for each participant in each learning condition. The participants were instructed to tell the experimenter as soon as they thought they had memorized all objects thoroughly. Then they took off the HMD, and the experimenter asked participants to name the colors of all nine objects, exemplified as hollow black circles printed on a white DIN A4 sheet of paper, in any order. If participants made an error, we granted them extra learning time within the virtual environment. This procedure was repeated until a participant had correctly reported the colors of all objects.

Afterward, participants carried out the corresponding set of test trials in the VR. We instructed them to perform this repositioning task as quickly and accurately as possible. After the learning and testing phases for either the horizontal or the vertical board orientation, the participants could take a short break before continuing with the respective other plane orientation, following the same principal procedure as before.

Before each learning phase, participants had ten practice trials using a different layout to get them familiar with the task. During these training trials we could address potential problems with color naming or control of the target object. After completing all tasks for both plane orientations, participants answered a questionnaire asking about their experience with VR and the strategies they used for memorization and reproduction during the experiment.

### Results and discussion

To control for outliers, we excluded trials on which the latency or angular error deviated more than three standard deviations from the respective overall mean (this affected 1.8% of all trials for latency and 3.7% for angular error). We analyzed the data in a 2 × 3 × 2 mixed-factors analysis of variance (ANOVA), with the within-subjects factors Plane Orientation (horizontal vs. vertical plane) and Test Orientation (0°, 180°, in-between board rotation), as well as the between-subjects factor Order (horizontal or vertical plane first), to investigate both of our hypotheses. If the ANOVA’s assumption of sphericity was violated in any of our experiments, we corrected the degrees of freedom using the Greenhouse–Geisser estimates. Follow-up contrasts were obtained using the methods implemented in lsmeans (Lenth, 2015). We only report effects of or interactions with the factor Order when they were significant.

**Fig. 2:**
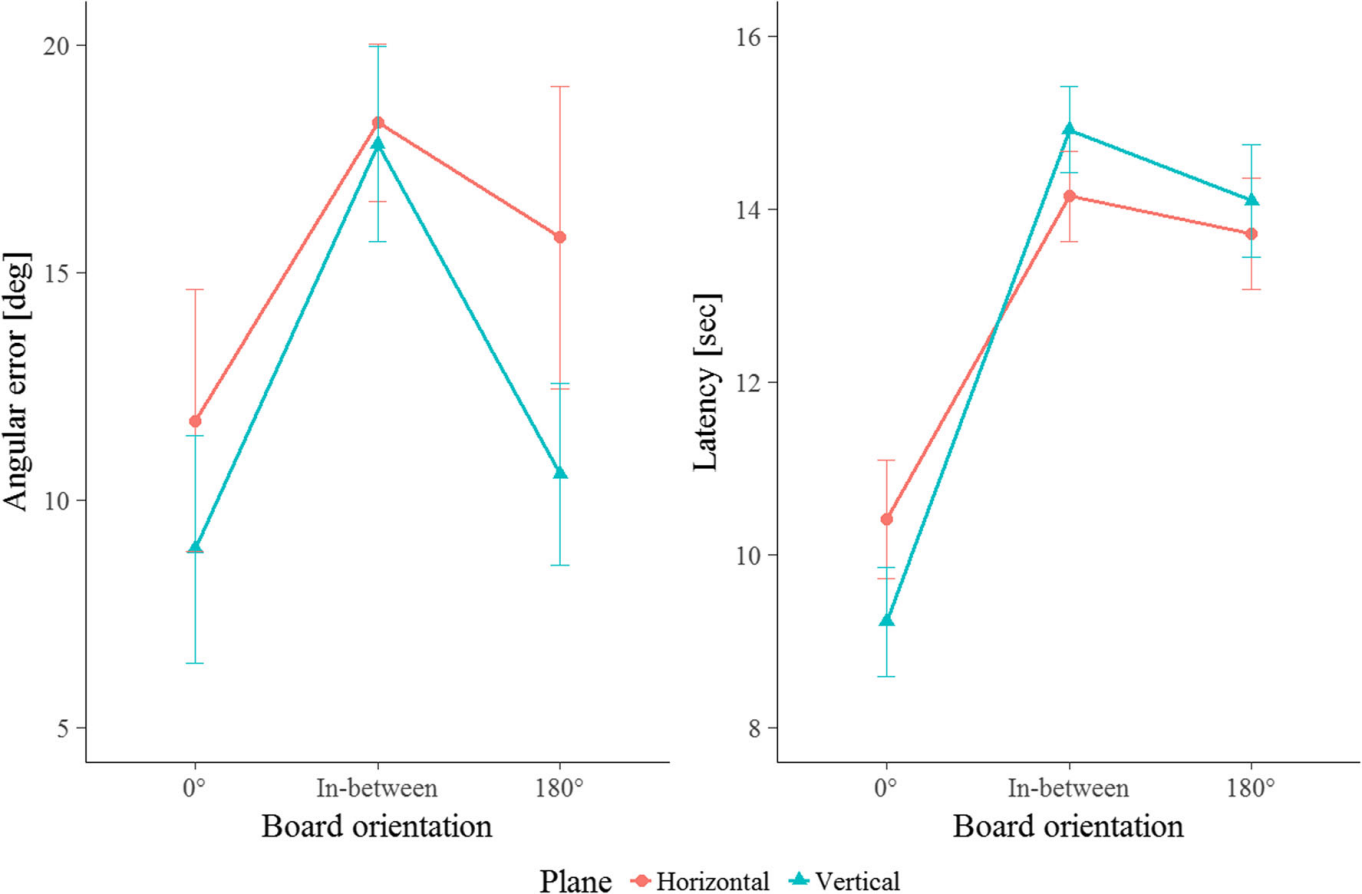
Angular error (left) and latency (right) in Experiment 1 as a function of test orientation: Experienced board orientation during learning (0°), the contra-aligned orientation (180°), and in-between orientations in both the horizontal (warm color) and vertical (cool color) planes. Error bars display standard errors of the means

The horizontal superiority hypothesis predicted better recall performance for object locations in the horizontal than in the vertical plane. As can be seen in Fig. 2 (and in Supplementary Fig. 1), for some test orientations the error pattern was numerically even opposite to this hypothesis. However, the ANOVA did not reveal any difference caused by plane orientation (horizontal vs. vertical) in terms of either error or latency, *F*s < 1.53, *p*s > .231, *η*_p_^2^ < .08. Thus, we found no support for the horizontal superiority hypothesis. General recall performance with horizontally distributed locations was not superior to performance with locations distributed in the vertical dimension, which is different from the findings obtained within multifloor spaces (Dolle et al., 2015; Hölscher et al., 2006; Luo et al., 2010; Montello & Pick, 1993; Thibault et al., 2013; Tlauka et al., 2007; Wilson et al., 2004). To test whether there was evidence in favor of the null hypothesis (i.e., that spatial memory was similar for the two planes), we examined the data by conducting a Bayesian *t* test, and therefore obtained Bayes factors using the Bayesian information criteria. The estimated Bayes factors were *BF*_01_ = 3.81 for error and *BF*_01_ = 3.42 for latency, indicating positive evidence (according to Raftery, 1995) in favor of the null hypothesis. If spatial relations have been learned from a single perspective, memory for horizontal and memory for vertical locations seem to yield similar performance.

The horizontal contra-aligned advantage hypothesis predicted a difference in recall patterns emerging with the contra-aligned layout orientation, in which this orientation is privileged only in the horizontal plane. As can be seen in Fig. 2, the performance pattern for neither of the two measures was in accordance with this hypothesis. The ANOVA revealed a main effect of test orientation on both error, *F*(2, 40) = 4.55, *p* = .017, *η*_p_^2^ = .19, and latency, *F*(1.51, 30.22) = 36.39, *p* < .001, *η*_p_^2^ = .65, indicating that recall performance varied across test orientations. However, there were no significant interactions between the factors Test Orientation and Plane, *F*s < 1.61, *p*s > .212, *η*_p_^2^ < .08, which is contrary to our predictions.

Because the omnibus test did not reveal a significant interaction, but solely a main effect of test orientation, we refrained from conducting detailed comparisons within each plane, but carried out contrasts comparing performance across test orientations after averaging over the factor Plane. These tests revealed that performance (in terms of both error and latency) for the orientation experienced during learning (0°) was significantly better than performance for the in-between orientations, *t*s < – 2.99, *p*s < .008, *d*s < – 0.91. With respect to error, the contra-aligned (180°) orientation did not differ from the 0° orientation, *t*(21) = – 1.10, *p* = .286, *d* = – 0.33, but by trend it did differ from the in-between orientations, *t*(21) = 1.89, *p* = .073, *d* = 0.57. In contrast, with respect to latency, a significantly lower response time was revealed when comparing the 0° with the contra-aligned test orientation, *t*(21) = – 6.81, *p* < .001, *d* = – 2.05, but no difference emerged when comparing the contra-aligned with the in-between orientations, *t*(21) = – 1.04, *p* = .308, *d* = – 0.31. Thus, whereas the contra-aligned orientation does not seem to be privileged in either of the spatial planes with respect to latency, with respect to error an advantage for the contra-aligned orientation over the in-between orientations is indicated for both planes.

For error, we obtained a significant interaction between the factors Plane Orientation and Order in which the learning conditions were carried out by the participants, *F*(1, 20) = 5.94, *p* = .024, *η*_p_^2^ = .23. This interaction revealed that participants made fewer errors in the condition they carried out second and suggests that participants performed better over time in general. With respect to latency, a trend toward a similar interaction was present, *F*(1, 20) = 4.08, *p* = .057, *η*_p_^2^ = .17.

Experiment 1 suggests that spatial memory for board locations learned from a single perspective does not differ between horizontal and vertical surfaces in either general performance level or recall patterns. These findings are in contradiction with the horizontal superiority hypothesis (targeting general performance level) and the horizontal contra-alignment advantage (targeting recall patterns). A Bayesian analysis even suggests similar processes, in terms of general performance level.

In Experiment 1 we tested participants with varying board rotations. Prior experiments reporting differences between horizontal and vertical memory have relied on participants or animals navigating through the environment (e.g., Hayman et al., 2011; Montello & Pick, 1993). This involves experiencing the space from different perspectives. Possibly, differences between horizontal and vertical memory can only be obtained if different perspectives are involved during testing. The predicted differences in recall from the contra-aligned orientation might emerge only with varying egocentric viewpoints on the board, seeing that the potentially underlying mechanism of spatial perspective taking during interpersonal interactions (Cavallo et al., 2017) involves whole perspective shifts. We aimed to test this possibility in Experiment 2.

## Experiment 2

In this experiment, we again tested our horizontal superiority and horizontal contra-aligned advantage hypotheses, but now in a setup in which participants were required to recall spatial relations from different positions in the room, and therefore from varying egocentric perspectives on the spatial layout.

### Method

#### Participants

Twenty-two naïve participants (15 women, seven men), ages 18 to 45 years (*M =* 27.18, *SD =* 6.64), were recruited from a participant database in exchange for monetary compensation. All participants gave written informed consent before the experiment.

#### Materials, design, and procedure

**Fig. 3:**
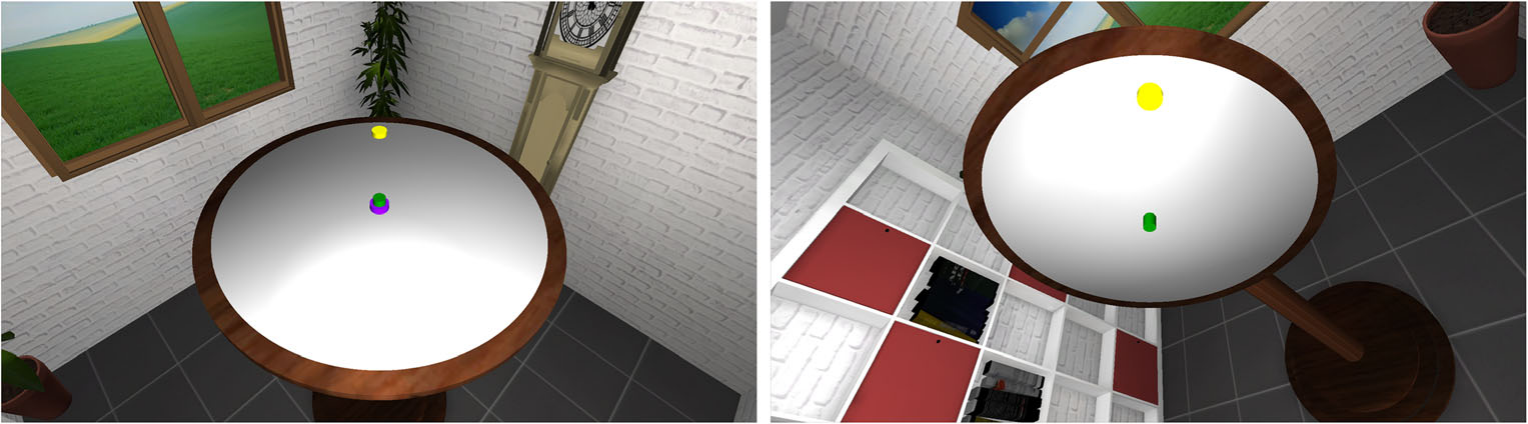
In Experiment 2 (as well as Exp. 3), the room orientations, and therefore the participant’s egocentric perspective on the board, varied across test trials (from 0° to 180°, in ± 45° steps). The present example corresponds to a room orientation of 45°. The learning phase remained identical to that in Experiment 1 (see Fig. 1, top row)

The materials, design, instructions, and procedure were identical to those of Experiment 1, except for the following: Whereas in Experiment 1 only the board orientation varied, here both the room and the board were rotated together by 0°, ± 45°, ± 90°, ± 135°, or 180° relative to the learning orientation (see Fig. 3). Thus, we varied the egocentric viewpoint onto the board.

### Results and discussion

The analysis was identical to that of Experiment 1. We considered 1.1% of the latency and 3.5% of the error data to be outliers and therefore excluded these data from the analysis.

**Fig. 4:**
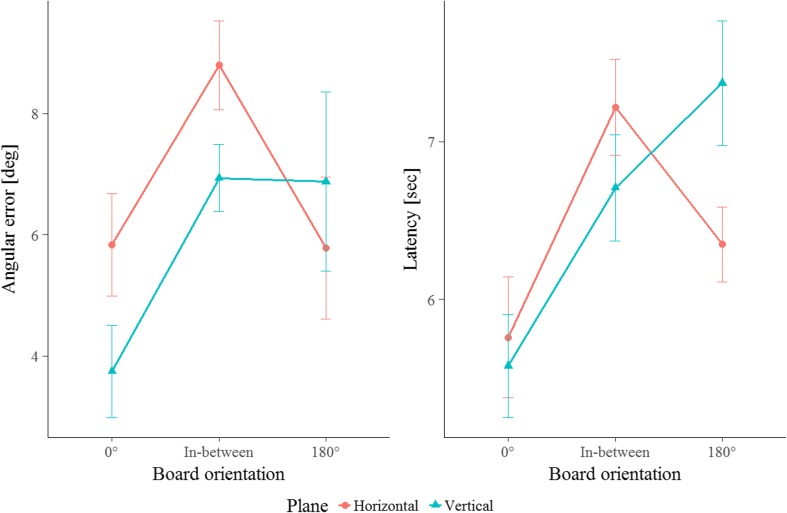
Angular error (left) and latency (right) in Experiment 2 as a function of test orientation (or viewpoint): Experienced room orientation during learning (0°), the contra-aligned orientation (180°), and in-between orientations in both the horizontal (warm color) and vertical (cool color) planes. Error bars display standard errors of the means

Again, our horizontal superiority hypothesis predicted better recall performance for object locations in the horizontal than in the vertical plane. As in Experiment 1, the performance patterns (shown in Fig. 4 and in Supplementary Fig. 2) were not in favor of this hypothesis, but rather point toward no or even an opposite effect. The repeated measures ANOVA did not reveal an advantage for one spatial plane over the other in terms of either error or latency, *F*s < 0.76, *p*s > .395, *η*_p_^2^ < .05. A Bayesian analyses revealed a Bayes factor of *BF*_01_ = 1.44 for error, and *BF*_01_ = 3.66 for latency, indicating weak or positive evidence in favor of the null hypothesis, respectively. These results corroborate the findings of Experiment 1 in rejecting the horizontal superiority hypothesis for locations learned from a single perspective, and extend them from tests with rotated layouts to tests with varying perspectives.

The horizontal contra-aligned advantage hypothesis predicted a difference in recall patterns emerging with the contra-aligned layout perspective, in which this orientation is privileged only in the horizontal plane. As can be seen in Fig. 4, the performance patterns are in accordance with this hypothesis. The ANOVA revealed significant main effects of test orientation on error, *F*(2, 40) = 8.34, *p* = .001, *η*_p_^2^ = .29, and on latency, *F*(1.52, 30.44) = 9.12, *p* = .002, *η*_p_^2^ = .31, indicating that performance varied across test orientations, as in Experiment 1. However, the main effect was qualified by an interaction with the factor Plane for latency, *F*(2, 40) = 5.50, *p* = .008, *η*_p_^2^ = .22, indicating that the recall patterns differed across planes. No such interaction was present with respect to error, *F*(1.42, 28.41) = 1.75, *p* = .206, *η*_p_^2^ = .08.

Due to the significant interaction effect on latency, we further analyzed the recall patterns to test our horizontal contra-aligned advantage statistically by testing for a difference across the factor Plane within the contra-aligned perspective and by conducting comparisons for every possible pair of test orientations in each spatial plane. Given that the plot for error in Fig. 4 (left plot) indicates a difference in performance patterns for horizontal and vertical locations that is in accordance with our horizontal contra-aligned advantage, we additionally conducted identical comparisons for error.

These comparisons revealed that within the contra-aligned test orientation, participants reacted more quickly in the horizontal than in the vertical plane, *t*(21) = – 2.28, *p* = .033, *d* = – 0.69. The numerical pattern was similar for errors, albeit not significant, *t*(21) = – 0.70, *p* = .490, *d* = – 0.21, suggesting that the difference was not due to a speed–accuracy trade-off.

In the vertical condition, performance for the 0° and contra-aligned test orientations did differ significantly, *t*s < – 2.54, *p*s < .020, *d*s < – 0.76. Performance for the 0° test orientation was significantly better than for the in-between test orientations, *t*s < – 2.59, *p*s < .018, *d*s < – 0.77. The contra-aligned orientation did not differ from the in-between orientations, *t*s > – 1.61, *p*s > .124, *d*s > –0 .49. Accordingly, memory recall from the contra-aligned vertical perspectives is not privileged as compared to other novel perspectives.

In contrast, in the horizontal condition performance (in terms of both error and latency) for the test orientation experienced during learning (0°) and for its contra-aligned (180°) orientation did not differ significantly, *t*s < 1.43, *p*s > .170, *d*s < 0.45. However, performance for both the 0° and the contra-aligned test orientations was significantly better than that for the in-between test orientations, *t*s < – 2.09, *p*s < .049, *d*s < – 0.62. These findings show that the contra-aligned perspective is privileged relative to other novel perspectives in the horizontal plane, which is in line with previous findings (McNamara et al., 2008; Meilinger & Bülthoff, 2013).

Regarding the factor Order, we obtained a significant interaction with the factor Plane Orientation during learning for latency, *F*(1, 20) = 5.58, *p* = .028, *η*_p_^2^ = .22. This interaction revealed that participants were quicker in the condition they carried out second.

The results concerning memory recall from varying egocentric viewpoints are in line with our horizontal contra-aligned advantage. Privileged memory recall for contra-aligned viewpoints seems to be unique for locations distributed in the horizontal plane. Because no such advantage appeared in Experiment 1 (which used varying layout rotations), retrieval with changing layout orientations might function differently from retrieving spatial relations from varying vertical viewpoints. In the latter scenario, humans apparently cannot benefit from the advantage of the contra-aligned perspective over other novel perspectives in the vertical plane. This might be due to the fact that humans do not adopt full-body vertical upside down perspectives naturally and also are not used to taking such viewpoints mentally (Waller, 2014). In contrast, humans frequently adopt different perspectives in the horizontal plane, since they naturally walk around in this plane and are used to mentally taking different horizontal perspectives. For instance, in conversations humans often take the perspective of their conversation partner (Mainwaring et al., 2003; Schober, 1993; Tversky et al., 1999), with the conversation partner being most frequently contra-aligned to oneself. Hence, humans have much more experience with contra-aligned perspectives in the horizontal than in the vertical plane. The lack of such experience within the vertical plane might lead to inefficient recall from contra-aligned perspectives in the vertical plane, as we observed. In Experiment 3, we intended to replicate these findings and to dig deeper into the underlying processes.

## Experiment 3

We conducted Experiment 3 to corroborate the findings of Experiment 2 with respect to the horizontal contra-alignment advantage hypothesis. Furthermore, we aimed to examine whether the underlying process consisted of a well-trained perspective shift due to repeated execution in the horizontal, but not the vertical, plane. To do so, participants repeatedly recalled board orientations that involved perspective shifts. Our prediction was that the presumably comparatively untrained 180° vertical shift would profit greatly from repetition, and more so than the already highly trained 180° horizontal shift. The patterns should become more similar over the course of testing.

### Method

#### Participants

Twenty-four naïve participants (13 women, 11 men), between 18 and 42 years of age (*M* = 25.21, *SD* = 4.97) participated in exchange for monetary compensation, and all participants gave written informed consent before the experiment.

#### Materials and procedure

The materials, design, instructions, and procedure were similar to those of Experiment 2. However, in addition to the viewpoint experienced during learning and its contra-aligned perspective, participants saw only one and not all the six of the possible in-between viewpoints (one of ± 45°, ± 90°, or ± 135°) during testing. We did this to limit the number of trials and the time the experiment would take to complete. We balanced the occurrence of the in-between perspectives, meaning that one orientation was used for four different participants, and two of these participants first learned the horizontal and the other two first learned the vertical layout. We assigned participants to one of the in-between perspectives before the experiment proper. In addition, for each learning condition, participants performed a series of four blocks of test trials, with 21 trials in each block and with all possible target objects occurring once for each perspective (leading to seven trials for each of the 0°, in-between, and 180° perspectives). After a test block, participants could take a short break and take off the HMD.

### Results and discussion

To test our hypotheses, we analyzed the data using a 2 × 4 × 3 × 2 mixed ANOVA with the within-subjects factors Plane Orientation (horizontal plane vs. vertical plane), Test Block (1–4), and Test Orientation (viewpoints on the board, 0°, 180°, in-between), as well as the between-subjects factor Order (horizontal or vertical plane first). We considered 1.5% of the latency and 2.4% of the error data to be outliers, and therefore excluded these data from the analysis.

The ANOVA did not reveal a general advantage for one spatial plane over the other (horizontal plane vs. vertical plane) in terms of either error or latency, *F*s < 0.86, *p*s > .365, *η*_p_^2^ < .05. Bayes factors of *BF*_01_ = 4.41 for error and *BF*_01_ = 3.93 for latency indicated positive evidence in favor of the null hypothesis, meaning that the performance levels were similar in horizontal and vertical testing. These results corroborate the findings of Experiments 1 and 2, showing that no general performance advantage of memory for horizontal over memory for vertical locations exists for spaces learned from a single perspective.

**Fig. 5:**
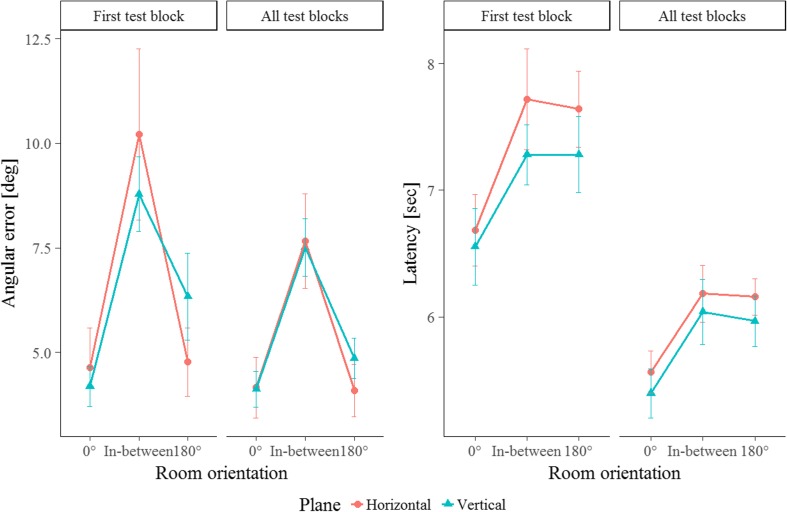
Angular error (left) and latency (right) for test trials of the first block and over all four blocks in Experiment 3 as a function of test orientation (or viewpoints): Experienced room orientation during learning (0°), the contra-aligned orientation (180°), and in-between orientations in both the horizontal (warm color) and vertical (cool color) planes. Error bars display standard errors of the means

The horizontal contra-aligned advantage hypothesis predicted a difference in recall patterns emerging with the contra-aligned layout orientation, where this orientation is privileged only in the horizontal plane. To be able to tell whether or not the results of Experiment 2 could be corroborated, the recall patterns for the first block of trials had to be investigated, as in this block the same numbers of trials were carried out for the 0° and 180° perspective. As is indicated by the left plot for each measured variable in Fig. 5, it appears that the performance patterns were not in accordance with our horizontal contra-aligned advantage and do not corroborate the findings of Experiment 2. The ANOVA revealed effects of test orientation on performance for both error, *F*(1.06, 23.35) = 11.83, *p* = .002, *η*_p_^2^ = .35, and latency, *F*(2, 44) = 9.38, *p* < .001, *η*_p_^2^ = .30, showing that recall performance varied across test orientations. However, the patterns were not different between planes—that is, no interaction between the factors Test Orientation and Plane, *F*s < 0.36, and no further (two- or three-way) interactions with the factor Test Block were observed, *F*s < 2.41. This suggests that the recall patterns were similar across both planes and blocks.

Due to the lack of any significant interactions, we refrained from detailed comparisons among a subset of the factor combinations for the first block of trials, but conducted contrasts to compare the performance across spatial planes averaged over the factors Plane and Test Block. These tests revealed that the 0° and 180° perspectives did not differ, *t*(23) = – 0.41, *p* = .684, *d* = – 0.17, but both led to significantly fewer errors than the in-between perspectives, *t*s < – 3.98, *p*s < .002, *d*s < – 1.61. In contrast, with respect to latency performance, the 0° perspective was significantly better than both the 180° and in-between perspectives, *t*s < – 3.49, *p*s < .003, *d*s < – 1.39, whereas the latter two did not differ from each other, *t*(23) = 0.46, *p* = .648, *d* = 0.19.

We could not replicate the results of Experiment 2 concerning the horizontal contra-alignment advantage. We did not observe any significant differences between planes in contra-aligned testing. For latency, participants were numerically even quicker in the vertical than in the horizontal case, which is opposite from the predicted direction. With respect to error, the contra-aligned perspective was privileged in both planes. Over the series of experiments, we therefore have no clear evidence for a difference in recall patterns emerging with the contra-aligned perspective.

In addition to the lack of an overall pattern difference between the planes, practice did not influence recall patterns differently in the two spatial planes. There was no interaction between the three factors of the ANOVA. We therefore did not find support for our hypothesis regarding a practice effect for the contra-aligned vertical perspective. This argues further against the horizontal contra-alignment advantage hypothesis. However, we did find main effects of test block for both error, *F*(1.85, 40.79) = 7.92, *p* = .002, *η*_p_^2^ = .26, and latency, *F*(1.79, 39.29) = 68.90, *p* < .001, *η*_p_^2^ = .76, showing that performance increased over the course of blocks in general.

As in the previous experiment, we obtained a significant interaction between the factors Plane Orientation During Learning and the Order in which these learning conditions were carried out by the participants for latency, *F*(1, 22) = 19.84, *p* = .001, *η*_p_^2^ = .47. Again, this interaction revealed that participants were quicker in the condition they performed second.

In addition to the analyses reported so far, we compared the general performance levels between the three experiments. The different scales of the ordinate axes in Figs. 2, 4, and 5 already suggest clear differences in errors and latencies between the first and the remaining two experiments. A mixed-factors ANOVA with the between-subjects factor Experiment and the within-subjects factor Plane (horizontal plane vs. vertical plane) corroborated this. It revealed strong effects of experiment in both error, *F*(2, 65) = 16.41, *p* < .001, *η*_p_^2^ = .34, and latency, *F*(2, 65) = 44.49, *p* < .001, *η*_p_^2^ = .58. No effects of or interactions with the factor Plane Orientation were present (*F*s < 1.10). Follow-up contrasts showed that performance was clearly better for Experiments 2 and 3 than for Experiment 1, *t*s > 4.45, *p*s < .001, *d*s > 1.08, but did not differ between Experiments 2 and 3 (*t*s < 1).

The clear performance advantages for perspective shifts in Experiments 2 and 3 over board-only rotations in Experiment 1 indicate that participants memorized relations between the object locations on the board and the surrounding room in long-term memory and used these relations to solve the configurational judgments. In Experiments 2 and 3 the board and room orientations were consistent during testing, such that participants could use both sources of information to determine the test orientation. In Experiment 1, the room and board orientations differed for nonexperienced test orientations (i.e., those other than 0°). Thus, participants had to rely on within-board information to infer their orientation, which was much harder to accomplish. If they had relied exclusively on object-to-object relations of the target objects, independently of the paradigm used, performance should have been equal in all experiments. The observed performance difference suggests that participants additionally memorized relations between the room and board locations and used them to solve the configurational task. This finding extends a popular model for configurational spatial memory, which states that exclusively object-to-object relations and the reference frame of their representation constitute the sole basis for configurational judgments (Mou, McNamara, Valiquette, & Rump, 2004). Our data indicate that these object-to-object relations also encompass relations to nontarget objects (here, room locations). One potential way of memorizing board and room locations together is an experienced view (for a discussion of how views might contain both self-to-object and object-to-object information, please see Meilinger & Vosgerau, 2010). Such a view will encompass not only the board itself, but also the surrounding room. In the case that the room and board locations do not match, only parts of the view can be used for the task, so performance deteriorates, as we observed. Similar findings indicating the use of background landmarks were found for recognizing an object layout on a table directly after presentation (Burgess, Spiers, & Paleologou, 2004; Mou, Xiao, & McNamara, 2008). Our findings extend these results from a recognition to a configurational task and from working memory to long-term memory retrieval.

Is it possible that participants used view-based memory only for self-localization, but not for the configurational judgments? We do not think so. To profit from self-localization in the judgment task, participants would still have had to know how the object layout on the board was related to the room, or they would not have been able to profit from the room information.

The performance advantage we found for perspective shifts over layout rotations also relates to previous findings obtained with imagined orientation changes. In a study of Wraga and colleagues, participants reacted more quickly and with fewer errors when they were required to imagine different egocentric viewpoints on a object configuration than when they imagined different rotations of the object layout itself while they remained at the same position (Wraga, Creem, & Proffitt, 2000). Since this viewer advantage disappeared for imagined rotations in vertical planes (Carpenter & Proffitt, 2001), it was suggested that the advantage occurs only if viewpoint rotations happen around the observers’ principal axes, where the body and the object layout are in an orthogonal relationship to each other, and may result from everyday experience with viewpoint changes in the egocentric horizontal plane (Creem, Wraga, & Proffitt, 2001). Our results extend the perspective-taking advantage from an imagined to a visual task, but indicate that such an effect might also be found with nonorthogonal body–layout relationships, as in the vertical plane in the present experiments.

## General discussion

We investigated whether memory of horizontal and vertical two-dimensional spatial locations differs when learned from one single view. Our results consistently showed similar performance levels in retrieving horizontal and vertical spatial memory in terms of both error and latency, in all three experiments. We observed quicker reactions in contra-aligned test perspectives for the horizontal than for the vertical plane in Experiment 2, but not so in the replication in Experiment 3. Furthermore, participants were clearly quicker and more accurate when tested from varying perspectives (Exps. 2 and 3) than with layout rotations (Exp. 1).

The first objective of the present work was to test whether vertical and horizontal memory differed with respect to performance level, as predicted by the horizontal superiority hypothesis. Previous work found superior spatial memory representations of horizontal than of vertical locations for human navigation in multi-level buildings (Dolle et al., 2015; Hölscher et al., 2006; Luo et al., 2010; Montello & Pick, 1993; Thibault et al., 2013; Tlauka et al., 2007; Wilson et al., 2004) and for neuronal coding within moving rodents (Hayman et al., 2011). Contrary to this horizontal superiority observed in memory acquired via navigation in large-scale environments, our results provide evidence for equal performance in accessing horizontal and vertical memory when the spatial relations were graspable from a single perspective. This result indicates that horizontal superiority is not a general property of spatial memory.

This does not necessarily contradict the bicoded-map hypothesis, which primarily targets spatial relations perceived through navigation and represented by entorhinal and hippocampal brain areas (Jeffery et al., 2013b). According to this hypothesis, self-motion cues are used in the process of computing spatial distances and directions in navigable spaces, with a higher resolution and therefore better memory for horizontal than vertical space. This memory can then be used for complex operations such as computing shortcuts, whereas stored information on large-scale vertical space may act instead as a contextual cue (see also Restat, Steck, Mochnatzki, & Mallot, 2004; Weisberg & Newcombe, 2013). Jeffery and colleagues further argued that for space perceived visually and without navigation, parietal regions are possibly involved, which might be capable of encoding spatial relations isotropically (Jeffery, Jovalekic, Verriotis, & Hayman, 2013a). Our findings support this idea: Similar processes are involved when learning spatial relations in different spatial dimensions visually and spatial asymmetries might relate primarily to spaces perceived via *locomotion*.

However, the spaces, learning conditions, and tasks used in experiments resulting in a horizontal advantage with navigable spaces versus our experiments differed not only in the necessity for locomotion, but also in several other respects, and some of these might very well also be responsible for the varying results. In our opinion (and in the insightful comments of the reviewers), reasonable candidates besides locomotion for explaining the pre- or absence of horizontal superiority include the size of the environment, the regularity of a spatial layout, two-dimensional versus three-dimensional spatial relations, the saliency of the planes involved, and the necessity of spatial integration. We discuss each of these candidates in the following paragraphs.

One factor modulating the presence of a horizontal advantage might be the *size* of the to-be-learned location layout, with an advantage emerging if the space is at a larger scale, where the interlocation distances and the distance to the observer would be of a greater magnitude than they were in the setup of the present study. Noticeably, this relates to locomotion, since very large spaces usually cannot be grasped from a single perspective, but must be experienced through navigation or different viewpoints. However, also in large-scale spaces perceived from a single perspective (e.g., in the mountains), horizontal locations might be encoded more accurately than vertical ones.

Besides, the *regularity* of a spatial location layout could play a role. For instance, previous experiments showed varying results on the structure of horizontal spatial memory as a function of layout orthogonality and symmetry (Richard & Waller, 2013). Conceivably, the grid-like pattern used in the present study might have helped participants to place the target objects more accurately within the correct location category (e.g., top-left or front-right). This might have concealed a horizontal advantage present in metric errors. Although this is a clear possibility, two arguments speak against it. First, when splitting the observed errors in the present experiment into categorical errors (angular deviation larger than 22.5°) and within-categorical metric errors (error smaller than 22.5°) we did not observe different results concerning the general horizontal advantage (we spare readers the statistical details). Second, one of the studies showing a horizontal advantage used rather regular building structures too (Thibault et al., 2013) suggesting that spatial asymmetry can also be observed with regular, grid-like layouts.

Moreover, the horizontal advantage might occur if spatial relations are scattered in *three-dimensional space* and not only on a two-dimensional plane. Increased difficulty when reasoning about spatial relations in three-dimensional versus two-dimensional spaces could trigger horizontal advantages. Since we used only two-dimensional planes in this study, both planes might have been treated equally, leading to a similar difficulty. However, the study of Thibault et al. (2013) also used a single vertical two-dimensional surface within a building and found variations in memory.

Another factor, which is related to the previous one, targets the *saliency of a plane*. Because a world-centered horizontal plane is orthogonal to gravity, it is rather easy to identify when locations are on the same horizontal plane. Contrarily, it is much more difficult for observers to identify within three-dimensional space whether locations are placed on the same vertical plane or not. This ambiguity for vertical space might lead to a disadvantage and the horizontal superiority might stem from this. Hence, the lack of the advantage in our experiments might be caused by the fact that horizontal and vertical locations were presented on a single plane, where it was apparent to the participants that the locations were placed on the same plane.

In addition, another potential factor is the *necessity or effort required for spatial integration*. In navigable spaces, spatial relations between locations can often not be grasped from a single perspective, but have to be derived from multiple views and their interconnecting trajectories (Meilinger, Strickrodt, & Bülthoff, 2016). Possibly, the previously observed horizontal advantage in buildings might be because the horizontal within-floor locations were closer connected and thus required less integration than the vertical between-floor locations. However, desktop experiments targeting exploration of multifloor buildings while controlling for within- and between-floor distances observed asymmetries nonetheless (Thibault et al., 2013). Alternatively, horizontal advantages might emerge due to more efficient and less error-prone integration across horizontal than across vertical views. This would also predict a horizontal advantage in a setup in which one must simply look around in space (e.g., turning from wall to wall, or plane to plane) to integrate spatial relations across different views. In the present experiments, no integration across views was required, and perhaps consequently, no advantage was observed.

In sum, the present experimentation showed that horizontal superiority is not a general property of spatial memory, but depends on the properties of the space and/or how it is learned. Multiple factors may constitute a required condition for horizontal superiority. Future studies should address these potential factors systemically by varying the need for locomotion, the size of the spatial layout, the layout regularity, the present spatial dimensions or planes as well as the extent to which spatial integration is required.

The second objective of the study was to test whether horizontal and vertical memory differed with respect to recall patterns, as predicted by the horizontal contra-alignment advantage hypothesis. We based this hypothesis on the fact that humans have much more experience with varying viewpoints and often take the spatial perspectives of other persons during interpersonal interactions like conversations (Cavallo et al., 2017; Mainwaring et al., 2003; Schober, 1993; Tversky et al., 1999) in the horizontal but not the vertical plane. However, although Experiment 2 showed some support for the horizontal contra-alignment advantage hypothesis, the effect was absent in the replication thereof in Experiment 3. Since the latter consisted of a greater number of test trials for the contra-aligned perspective, we regard it as more reliable, and therefore opt for rejecting the horizontal contra-aligned advantage hypothesis rather than supporting it. Thus, in comparison with retrieval of vertical spatial memory, experience with frequently taking a contra-aligned perspective in the horizontal plane seems not to necessarily privilege memory recall from such a perspective.

Furthermore, when considering the results pattern for error in Experiment 3 as well as the Supplementary figures, it seems that humans can benefit from the orthogonality and symmetry (e.g., Richard & Waller, 2013) of a vertical layout in a similar way to how they do in horizontal layouts, leading to better performance for contra-aligned (180°) and orthogonal orientations (± 90°; as suggested by the Supplementary figures) than for oblique orientations (± 45°, ± 135°). Possibly, these orthogonality/symmetry effects might stem from the relative ease of transforming the encoding perspective for recall between aligned orientations (e.g., by reversing/mirroring the memorized perspective), as was previously shown with a horizontal object layout (Street & Wang, 2014). Our findings suggest that participants might have applied and benefited from similar strategies in the vertical plane, even though they do not have extensive experience with vertical perspective shifts.

Moreover, we found a clear performance advantage for perspective shifts in Experiments 2 and 3 over the board-only rotations in Experiment 1. It indicates that in addition to relations within an object layout (Mou et al., 2004), people memorize the surrounding room as well as layout-to-room relations and use these relations to solve configurational judgments. Potentially, this memory representation was formed on the basis of the view experienced during learning. These view-based representations might encompass the same type of information independently of the spatial plane orientation of the to-be-memorized spatial configuration. If so, such a process might explain why similar results for the two spatial planes were found in our study. For both spatial planes, a representation in the form of a visual view (e.g., Christou & Bülthoff, 1999; Christou, Tjan, & Bülthoff, 2003) may be encoded and retrieved similarly.

Previous studies that used a perspective change paradigm often asked participants to perform judgments of relative direction (JRD; e.g., Kelly & McNamara, 2008; Meilinger & Bülthoff, 2013; Mou & McNamara, 2002; Shelton & McNamara, 2001). Like in our study, they first learned a configuration of objects, but during the test, participants did not receive any visual information, but had to imagine standing at a certain object and point to another location, while being situated in a novel environment. Consequently, the actual standing position of the participant will have little to no influence on the recall performance from varying imagined orientations of the learned environment. In contrast, if one remains in the learning environment, the current orientation will likely affect memory recall, with an increase in performance if the actual and the to-be-imagined orientations match (e.g., May, 2004). Because the participants remained in the environment in our visual perspective change setup, it is conceivable that a similar effect or conflict might have occurred. Although this was not an issue in Experiments 2 and 3, since the effects were equal for all test scenarios in these experiments, a significant conflict might have occurred in Experiment 1, in which participants remained at the position experienced during learning for all test conditions. However, as this potential conflict was similar for both spatial plane orientations, the interpretation of our results concerning the tested hypotheses should not be challenged by it. Nonetheless, it is an open question whether we would find similar results concerning spatial memory of horizontal and vertical locations with a JRD task. A previous study using a task with visual information, in which the layout had to be recognized from varying perspectives (as in the present study), showed recall patterns similar to those when using a JRD task (Meilinger & Bülthoff, 2013). However, if we bear in mind that Waller (2014) reported informally that participants struggle to perform the JRD task with upside-down perspectives, one might expect better performance in horizontal than in vertical JRD tasks. Potentially, such a result might then rather stem from a conflict between gravity and the imagined upside down body positions in vertical JRD tasks, which are not present in horizontal JRD tasks. These results might therefore not target the quality of spatial memory itself, but rather the required processes during retrieval.

We recognize that the reported null effects and their interpretation concerning the horizontal superiority hypothesis can be a troublesome prospect. However, on the basis of the Bayesian analyses we conducted and the replication of the evidence for the null effect across three experiments, we think we have created a strong enough case against the hypothesis that horizontal superiority is a general property of spatial memory. We would further back up this case by arguing that our setup was sensitive enough to detect other meaningful results described in the literature, such as better memory recall from experienced than from novel test orientations, as well as the novel result concerning the difference between the first and the latter two experiments. Consequently, we think that the interpretation of the null effects is valid.

In conclusion, contrary to findings obtained with spaces learned through navigation, two-dimensional spatial arrangements learned from a single perspective did not show an advantage for horizontal over vertical memory, but were similar to each other in terms of memory retrieval performance. Vertical and horizontal memories not only showed similar overall performance, but also exhibited similar dependencies on perspective change. We observed no robust differences in the recall patterns when recalling spatial relations from multiple test orientations. Finally, our results imply that spatial memory is not confined to relations between target locations, but also incorporates relations between the target objects and the surrounding room—for example, in the form of a memorized view. Spatial memory thus does not differ per se as a function of spatial plane orientation, but variations might originate instead from the way the space was structured and experienced initially.

## Electronic supplementary material

ESM 1 (DOCX 185 kb)
