# Supplementary material for: No advantage for remembering horizontal over vertical spatial locations learned from a single viewpoint
Source: Mem Cognit. 2017 Sep 5;46(1):158–71. doi: 10.3758/s13421-017-0753-9 (PMC5758721; doi:10.3758/s13421-017-0753-9)
Supplement: Supplementary file 1 — (DOCX 185 kb) [file 13421_2017_753_MOESM1_ESM.docx]

# Supplementary Materials


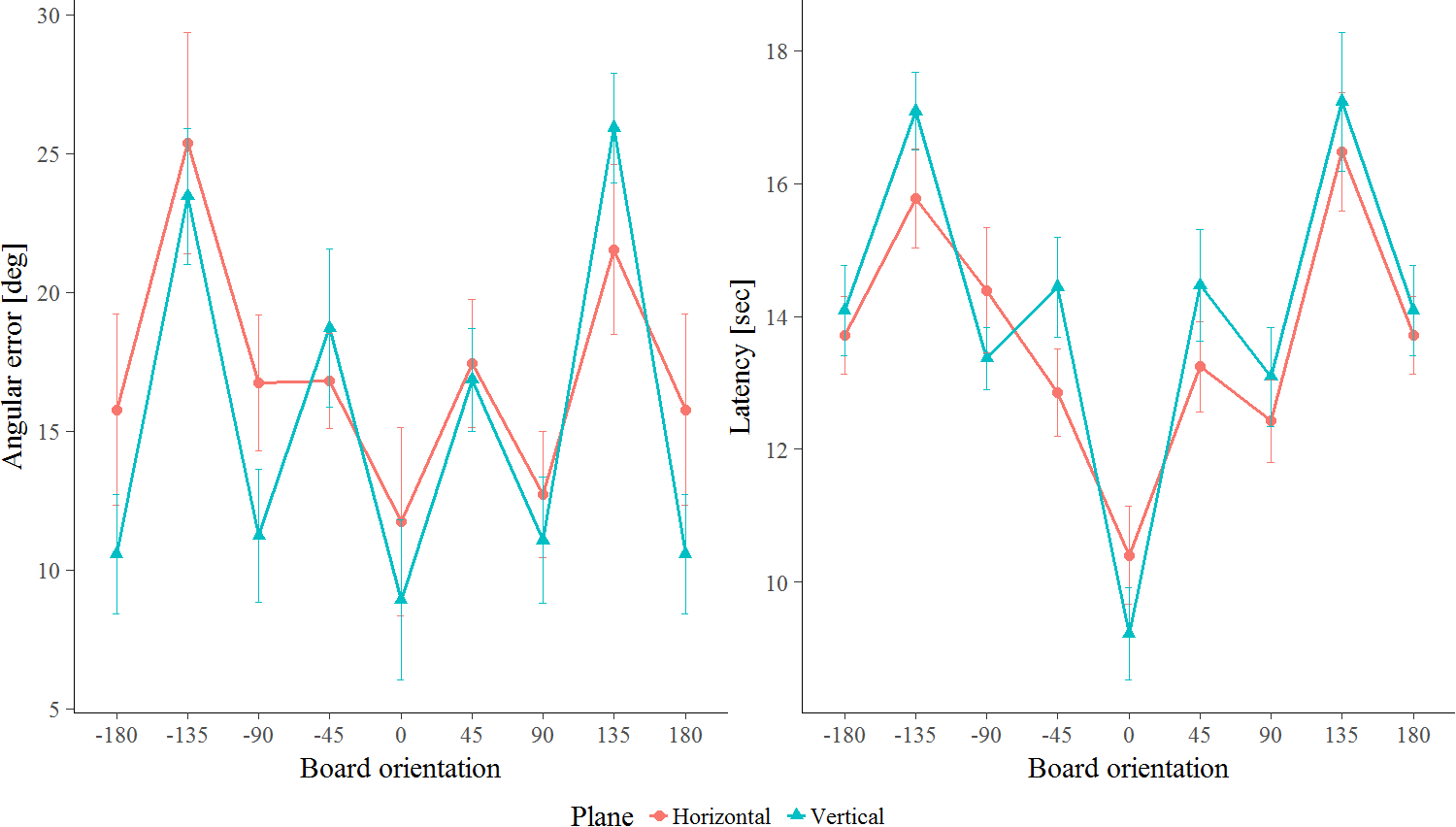


*Supplementary Figure 1*. Angular error (left) and latency (right) in Experiment 1 as a function of board orientation (from 0° to ±180°, in ±45° steps) in the horizontal (red) and vertical (blue) plane. Values for the -180° and 180° directions are identical. Error bars display standard errors of the mean.


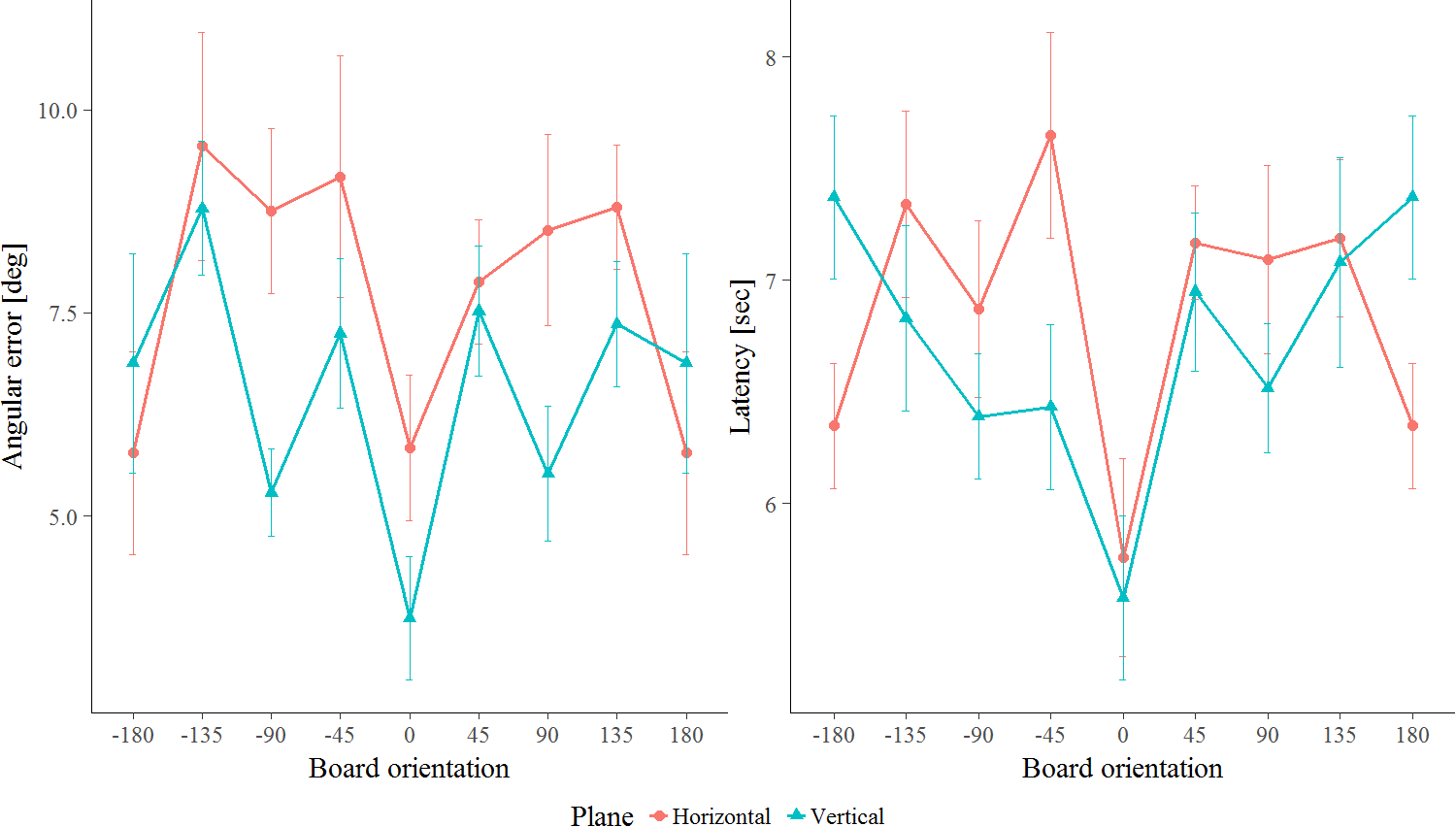


*Supplementary Figure 2*. Angular error (left) and latency (right) in Experiment 2 as a function of room orientation (from 0° to ±180°, in ±45° steps) in the horizontal (red) and vertical (blue) plane. Values for the -180° and 180° directions are identical. Error bars display standard errors of the mean.
